# Pancreatic acinar cystadenoma in a background of diffuse multifocal pancreatic cystic lesions: A case report

**DOI:** 10.1016/j.ijscr.2020.07.026

**Published:** 2020-07-15

**Authors:** Mohammed Abdulrahman Alkhateeb, Deena Boqari, Nabeel Khalid Mansi

**Affiliations:** a Imam Abdulrahman Bin Faisal University, Dammam, Saudi Arabia; b Department of Pathology, King Fahad Specialist Hospital, Dammam, Saudi Arabia; c Department of Surgery, King Fahad Specialist Hospital, Dammam, Saudi Arabia

**Keywords:** Acinar cell cystadenoma (ACA), Neoplasm, Diffuse, Intraductal papillary mucinous neoplasm (IPMN), Case report

## Abstract

**Introduction:**

Acinar cell cystadenoma (ACA) is very rare and benign neoplasm of the pancreas; it arises from the normal acinar parenchyma of the pancreas and may exist as solitary or diffuse lesions.

**Presentation of case:**

We here present a case of thirty-seven years old gentleman who incidentally was found to have multifocal cystic lesions distributed throughout the tail, body and uncinate process of the pancreas.

**Discussion:**

Pre-operative diagnosis of pancreatic ACA remains very difficult and challenging despite advances in imaging techniques. Due to diffuse involvement of the pancreas by different size cystic lesions in this case, the initial diagnosis was suspected to be SB-IPMNs. A Surgical resection was performed, and pathological analysis of the lesions was consistent with ACAs.

**Conclusion:**

To avoid unnecessary major pancreatic resection and its consequent complications. We hereby recommend clinicians to have a high index of suspicion for ACA within the differential of pancreatic cystic neoplasm.

## Highlights

• Mimickers of pancreatic side branch Intraductal Papillary Mucinous Neoplasm.
• Distinguishing between neoplastic and non-neoplastic pancreatic cystic lesions.
• The natural history of pancreatic acinar cystadenoma: Is it a pre-cancerous lesion?
• Acinar cystadenoma prognosis appears to be good even if it is incompletely resected.
• High index of suspicion for Acinar cystadenoma in pancreatic cystic lesions.

## 1 Introduction

A Major part of the pancreas is made up of Acinar cells, yet pancreatic neoplasms exhibiting acinar differentiation are very uncommon, two known types exist: acinar cell cystadenocarcinoma and acinar cell cystadenoma (ACA). They represent not more than 2% of all the neoplasms of the pancreas [1]. ACA is a very rare and benign epithelial cystic growth that usually arises from the normal acinar parenchyma of the pancreas. It occurs predominantly in women [2]. The deducted age of onset ranges between 9 and 71 years with a mean age of 42.9 years [3]. The first case was described by Albores-Saavedra in 2002 as an autopsied case [4]. ACA can exist as a multilocular or unilocular lesion and mostly solitary. However, they rarely present as a diffuse disease which is described in less than 10% of all reported cases of ACAs [3]. Patients usually present with abdominal pain though owing to the increasing use of Cross-sectional imaging, Incidental diagnosis is becoming more common. There has been no malignant transformation observed or published in the literature as of writing this. As a result of the insufficient number of cases reported, the natural course is yet uncertain. In this report, we describe a case of a healthy adolescent male presenting with this rare cystic lesion. This work has been reported in line with the SCARE criteria [5].

## 2 Case presentation

**Fig. 1:**
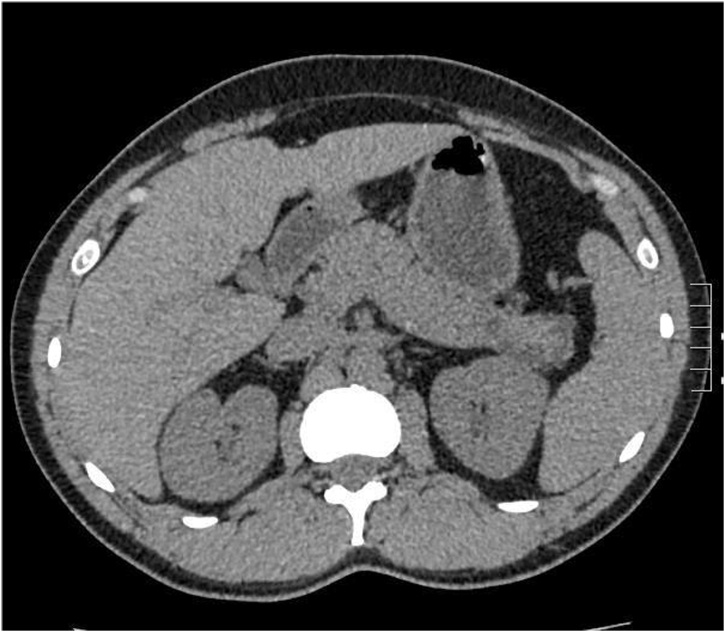
Non contrast CT scan of the abdomen of the shows few cystic lesions as the tail of pancreas.

**Fig. 2:**
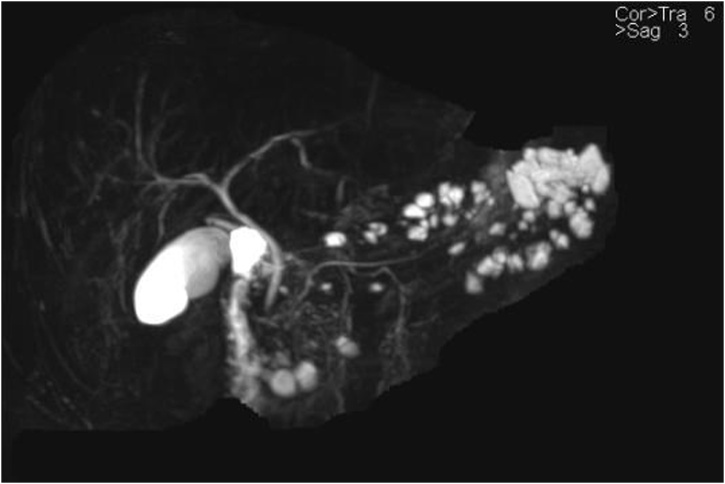
MRI of the pancreas shows diffuse multifocal cystic lesions confined to body-tail and uncinate prosses of the pancreas with no communication to PD.

**Fig. 3:**
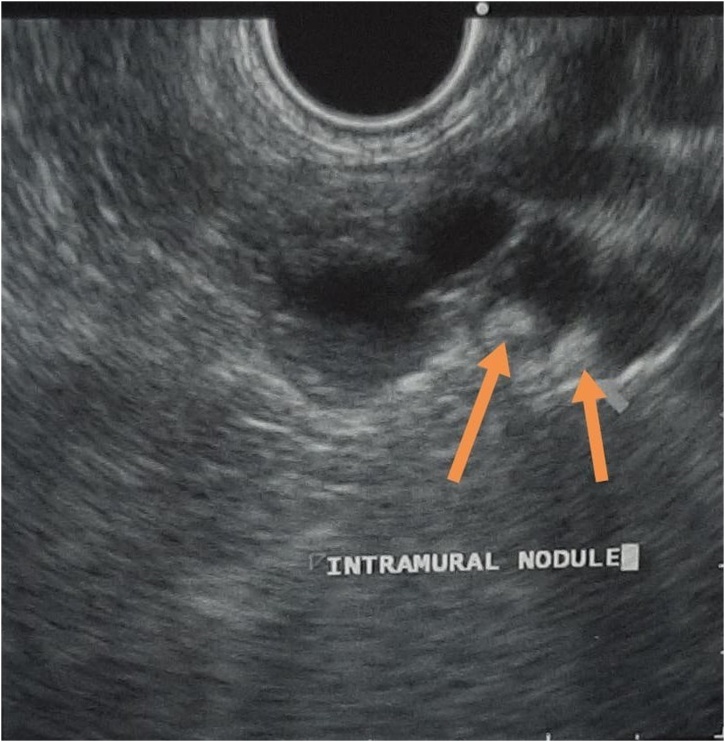
EUS of the pancreas shows body-tail cystic lesions with 2 intramural nodules.

A 37-year-old gentleman had been incidentally found to have a diffuse multifocal cystic lesions of the pancreas at a former hospital, it had been pointed out by a screening computed tomography (CT) scan to investigate a nonspecific abdominal pain (Fig. 1). After he was transferred to our hospital, a baseline magnetic resonance imaging (MRI) was performed, which revealed multifocal cystic lesions confined to the uncinate process, body, and tail of pancreas associated with no high-risk stigmata neither a worrisome feature (Fig. 2). A multifocal Branch duct intraductal papillary mucinous neoplasm (BD-IPMN) was considered as a main differential diagnosis. Then, a routine follow-up has been provided to put the lesions under surveillance. Two years after the diagnosis he had an Endoscopic ultrasound sonography (EUS) which showed a 2.2 cm cystic lesion in the body-tail of pancreas associated with septation and Intramural nodules with no clear communication with the main pancreatic duct (PD) (Fig. 3). PD size is normal in body and tail. During same a sample was obtained for cytology. It yielded a positive result for cellular atypia and mucin. He had no familial history, past medical history. Physical examination was unremarkable. The laboratory data was almost normal including pancreatic tumor markers.

BD-IPMN suspicious for malignant transformation was considered as a provisional diagnosed. According to pancreatic cystic lesions guidelines [6]. Surgical resection might be recommended because of his age, the cytology result and the presence of intramural nodules. Therefore, we decided to propose surgical resection and written informed consent was obtained. We decided that it is reasonable to perform a segmental resection to remove the IPMNs with the highest oncological risk at body-tail of pancreas and perform surveillance of the remaining lesions at the uncinate process.

Intraoperatively, we found multifocal cystic lesions at the tail-body of pancreas without invasion to the adjacent tissues. Due to the possibility of transformed IPMN, we performed a distal pancreatectomy, splenectomy, and a systemic lymph node dissection. The postoperative course was uneventful.

**Fig. 4:**
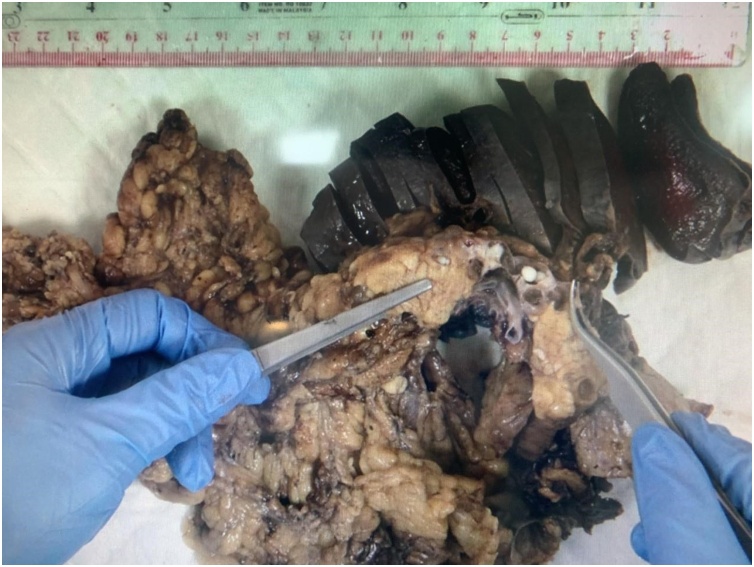
Gross specimen of the distal pancreas and spleen shows body and multilocular cystic lesions body-tail of the pancreas filled with whitish egg like material.

In pathological examinations, sectioning of the pancreas revealed multicystic lesions. The Cysts are filled with small white rounded firm egg-like material, the largest cyst measures 3.0 × 3.0 × 1.4 cm. The spleen was unremarkable (Fig. 4).

**Fig. 5:**
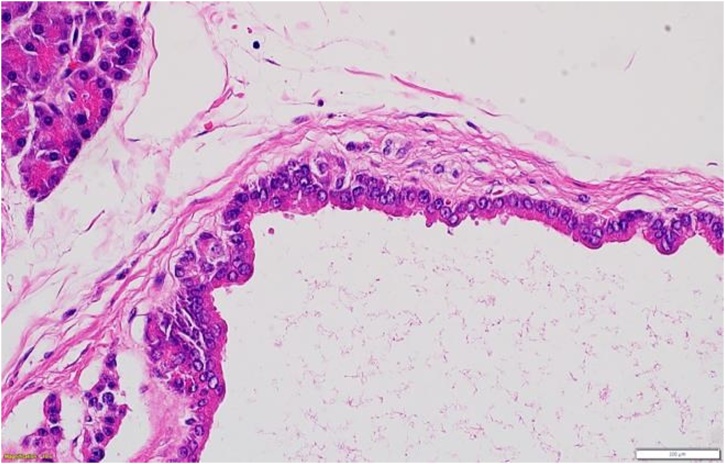
Single layer of cuboidal acinar epithelium with apical eosinophilic zymogen granules with uniform basally located nuclei (Hematoxylin-eosin, original magnification ×400).

**Figs. 6 and 7:**
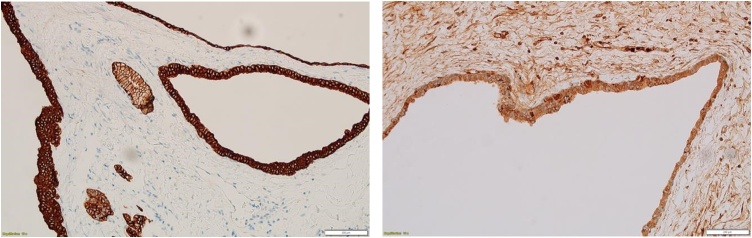
Diffuse cytoplasmic and membranous expression of CK8/18 and trypsin (Immunohistochemistry, original magnification ×200).

Histologically, the hematoxylin and eosin sections showed lobules of pancreatic parenchyma with multiloculated variably sized thin-walled cysts, some contain dense inspissated eosinophilic laminated concretions. They are lined by a single layer of cuboidal acinar epithelium with apical eosinophilic zymogen granules with uniform basally located nuclei (Fig. 5). By Immunohistochemistry, the acinar cells showed strong cytoplasmic and membranous reactivity for CK8/18, trypsin, and chymotrypsin which confirmed the acinar cell differentiation (Figs. 6 and 7).

The final diagnosis was pancreatic acinar cystadenoma. Nine months have passed after resection, and no tumor recurrence and symptoms were observed.

## 3 Discussion

Acinar cell neoplasms are recognized as a rare entity. Particularly, ACAs, lined by cells with acinar differentiation without atypical changes, are reported to be extremely rare [7]. ACA incidentally detected during autopsy was first reported in April 2002. After that, several case reports have been reported, and eventually, the definition of ACA was established by the WHO classification of tumors of the digestive system in 2010 [8].

In a literature review done in 2016 identifying 48 cases [3], females were predominant. Approximately two-thirds of the patients had complained of abdominal pain or discomfort. The postoperative follow-up period was widely ranged from 5 months to 11 years. There has not been any case reported on recurrence or evident malignant transformation so far. All the reviewed patients were diagnosed by the pathological findings of surgical or autopsied specimens.

The preoperative diagnosis of ACA is usually based on a combination of clinical features, radiological characteristics, and serological findings. In some ACAs, the cystic lesions might cause clinical symptoms, while others are incidental findings on abdominal imaging. Although of crucially clinical importance, accurate preoperative diagnosis is difficult or even impossible because ACAs have less definitive morphologic features in comparison to other pancreatic cystic lesions.

In our case, there is diffuse involvement of the pancreas by different size cystic lesions scattered throughout the whole pancreas. Due to diffuse nature of the disease in radiological finding the initial diagnoses was suspected to be SB-IPMNs of pancreas. Also, there were no features suggestive of high-risk stigmata neither worrisome features [6]. Therefore, we elected to manage our patient conservatively by subjecting him to surveillance imaging. Eventually, after two years a EUS has been performed which showed one of the cystic lesions in the body-tail of pancreas has a septation and Intramural nodules. Moreover, Cellular atypia and mucin were found in the cytology sample. Thus, a provisional diagnosis of BD-IPMN in association with malignant transformation was made. At this stage, a decision is made to perform a distal pancreatectomy and splenectomy to remove the IPMNs with the highest oncological risk. The rest of the lesions at the uncinate process were subjected to surveillance as they were lacking worrisome features.

The final pathology report confirmed the absence of malignancy or dysplasia but interestingly, the lesion was found to be ACA. Additionally, further sections and samples from the pathology specimen were examined which ruled out a coexisting SB IPMN as it was observed in one of the reported cases in the literature [9]. Accordingly, we presumed that the remnant lesions in the uncinate process would most likely represent ACA. Up to date, there is no evidence that ACAs are pre-malignant neoplasm and hence no further surgical intervention is required at the moment. Besides if the lesions turn up to be SB IPMNs, to the latest guidelines there is no need for surgical resection in the absence of high-risk stigmata and worrisome features. Thus, completion pancreatectomy was avoided in this case.

To the moment, the patient is asymptomatic, he has a normal biochemical profile including blood sugar measurements and has stable lesions in recent imaging.

## 4 Conclusion

ACAs are rare pancreatic neoplasms and are very difficult to be diagnosed preoperatively. Similarity to other pancreatic cystic neoplasms makes this diagnosis even more challenging. In this report, we described diffuse multifocal pancreatic cystic lesions which turn out to be ACA after conservative pancreatic resection. With regards to the remnant lesions in the residual part of pancreas, they would most likely represent ACAs. Due to lack of evidence of malignant transformation of ACAs, a decision is made not to subject the patient to completion pancreatectomy. We decided to publish this case to highlight the importance of having a high index of suspicion for ACAs when treating pancreatic cystic neoplasm. Also, a frozen section biopsy might be taken in account when facing such case. This will allow the pancreatic surgeon to avoid performing unnecessary major pancreatic resection such as a whipple procedure or total pancreatectomy that may lead to significant morbidities including exocrine dysfunctions, brittle diabetes, and poor quality of life.

## Declaration of Competing Interest

No Personal relationships with, or ﬁnancial interest in, any commercial companies pertaining to this article.

## Sources of funding

This article was self-funded.

## Ethical approval

This case was approved by KFSH ethical committee.

## Consent

Written informed Consent was obtained and signed by the patient.

## Author contribution

1. Mohammed Abdulrahman ALkhateeb: Data collection, Data analysis, ًWriting the manuscript, Review.
2. Deena Boqari: Pathological analysis and report.
3. Nabeel Khalid Mansi: Study concept, Study design, Data collection, Data analysis, Writing the manuscript, Review.

## Research studies

This is not a first in man case report. So it can’t be registered in the research registry.

## Guarantor

Nabeel Khalid Mansi.

## Provenance and peer review

Not commissioned, externally peer-reviewed.
